# Development of an interpretable machine learning model associated with genetic indicators to identify *Yin*-deficiency constitution

**DOI:** 10.1186/s13020-024-00941-x

**Published:** 2024-05-15

**Authors:** Jing Li, Yingying Zhai, Yanqi Cao, Yifan Xia, Ruoxi Yu

**Affiliations:** 1 National Institute of Traditional Chinese Medicine Constitution and Preventive Medicine, Beijing University of Chinese Medicine, No.11, Bei San Huan Dong Road, Chaoyang District, Beijing, 100029 China; 2 School of Chinese Medicine, Beijing University of Chinese Medicine, Beijing, 100029 China

**Keywords:** Traditional Chinese medicine, *Yin*-deficiency constitution, Constitution identification, Machine learning, Prediction model, Model interpretation

## Abstract

**Background:**

Traditional Chinese Medicine (TCM) defines constitutions which are relevant to corresponding diseases among people. As one of the common constitutions, *Yin*-deficiency constitution influences a number of Chinese population in the disease onset. Therefore, accurate *Yin*-deficiency constitution identification is significant for disease prevention and treatment.

**Methods:**

In this study, we collected participants with *Yin*-deficiency constitution and balanced constitution, separately. The least absolute shrinkage and selection operator (LASSO) and logistic regression were used to analyze genetic predictors. Four machine learning models for *Yin*-deficiency constitution classification with multiple combined genetic indicators were integrated to analyze and identify the optimal model and features. The Shapley Additive exPlanations (SHAP) interpretation was developed for model explanation.

**Results:**

The results showed that, NFKBIA, BCL2A1 and CCL4 were the most associated genetic indicators with *Yin*-deficiency constitution. Random forest with three genetic predictors including NFKBIA, BCL2A1 and CCL4 was the optimal model, area under curve (AUC): 0.937 (95% CI 0.844–1.000), sensitivity: 0.870, specificity: 0.900. The SHAP method provided an intuitive explanation of risk leading to individual predictions.

**Conclusion:**

We constructed a *Yin*-deficiency constitution classification model based on machine learning and explained it with the SHAP method, providing an objective *Yin*-deficiency constitution identification system in TCM and the guidance for clinicians.

## Background

In modern medical research, clinicians and researchers have gradually paid attention to individual-concerned approaches instead of disease-concerned approaches. Individualized medical care has gained more and more attention in contemporary medicine [1]. Traditional Chinese Medicine (TCM) constitution prioritizes constitution identification, emphasizing people-oriented approaches in diagnosing and treating diseases. This idea focuses on individual differences and provides effective methodologies for individualized treatment from a TCM perspective [2]. TCM constitution is a theoretical system that guides the prevention, treatment of diseases and the rehabilitation of health, through studying the characteristics of the nine basic constitution types and their association with diseases. The theory posits that different diseases may share a common fundamental constitution. TCM constitution utilizes modular approaches to divide the population into nine major categories including a balanced constitution and eight biased constitutions (*Qi*-deficiency, *Yang*-deficiency, *Yin*-deficiency, Phlegm-dampness, Damp-heat, Blood-stasis, *Qi*-stagnation, and Special constitution) [3]. Constitution types can influence the onset and progression of a disease. Different constitution types have different susceptibility to pathogenic factors and propensity for disease onset [4]. The TCM constitution theory is based on disease research, prevention, and treatment, emphasizing individualization and systematization. This expands the concept of individualized diagnosis and treatment in modern medicine.

An epidemiological survey on TCM constitution types showed that *Yin*-deficiency constitution (YinDC) was the most common constitution among all the constitutions in the elderly population over 65 years old [5]. YinDC is one of the most common constitutions, accounting for about 8.27% of the total Chinese population [6]. YinDC is caused by a deficiency of yin-fluid and mainly characterized by deficiency-heat manifestations such as dry mouth, feverish palms and soles [7]. The clinical evidence shows that the biased YinDC is related to the occurrence of many common diseases. Studies showed that YinDC is the most distributed constitution type among patients with hypertension, diabetes, constipation, menopausal syndrome, and insomnia, et al. [8]. *Yin*-deficiency related diseases can be effectively prevented by regulating the YinDC [9]. Therefore, identifying YinDC accurately is crucial for preventing and treating *Yin*-deficiency related diseases.

Researchers have developed some mature methods to identify YinDC. Nowadays, the Constitution in Chinese Medicine Questionnaire (CCMC) is widely used in the clinical identification of YinDC [10]. Besides, clinicians also diagnose YinDC using the traditional four TCM diagnostic methods. However, these two identification methods solely rely on self-reported symptoms and the experience of clinicians. The diagnose results may be affected by patients’ abnormal input types in the questionnaire or doctors’ subjectivity bias when collecting information from the four diagnostic methods [11]. Therefore, introducing modern technology into the YinDC identification is important to improve the accuracy of constitution identification. Artificial intelligence (AI) is one of the most prominent modern technology. With the growing demand for constitution identification, several AI techniques have been applied to TCM constitution identification research [12]. Machine learning (ML), a branch of AI, is an emerging technique for discovering functional patterns through complex algorithms on large-scale heterogeneous data sets. Compared to manual analysis, its powerful algorithms enhance the efficiency and reliability of data analysis and prediction [13]. Currently, machine learning is integrated with infrared thermographic testing [14], tongue image automatic identification [15], pulse waveform mapping [16], and modern acoustics [17] to analysis somatic characterization information of constitutions for identification. However, these techniques for constitution identification rely on collecting macroscopic representational information from the human body. These methods may also be compromised due to the restriction of equipment conditions and the interference of external factors such as image acquisition angle and brightness, which ultimately reduces the reliability of constitution identification methods [18]. Thus, obtaining more objective microscopic diagnostic information is essential to improve the accuracy of YinDC identification using machine learning.

Genetic indicators are objective microscopic information that is extremely valuable for precise, objective diagnosis of diseases [19]. Relevant studies have shown that there were differences in gene expression between YinDC and balanced constitution (BC) [20]. Five genes, TGF beta-activated kinase 1 (TAK1), NFKB inhibitor alpha (NFKBIA), Chemokine CC motif ligand 4 (CCL4), BCL2 related protein A1 (BCL2A1), and Interleukin-8 (IL-8), in the NF-κB signaling pathway related to inflammation and aging are abnormally expressed in YinDC [21]. Thus, these five indicators are essential references for identifying YinDC. To our knowledge, there were no approaches to identify YinDC with genetic indicators detection in existing research. To improve the objectivity, accuracy, and efficiency of YinDC identification, it is essential to establish a predictive model based on the specific genetic indicators of YinDC by machine learning.

A previous study examined the expression levels of TAK1, NFKBIA, CCL4, BCL2A1, and IL-8 in participants’ blood to identify the different gene expression of females with YinDC and BC [22]. Based on the previous discovery, we used the five genetic indicators and applied four machine learning methods to construct predictive models of YinDC. Four machine learning methods were used including logistic regression, random forest, support vector machine (SVM) [23], and eXtreme Gradient Boosting (XGBoost) [24]. Furthermore, we used the Shapley Additive exPlanations (SHAP) interpretation tool [25] to provide an intuitive interpretation of the predictive models. This tool helps with explicit interpretations of individualized risk predictions and allows clinicians to visualize the impact of critical features in the models [26].

This paper established predictive models of YinDC by using machine learning methods based on five genetic indicators, providing accurate and efficient technologies for clinical YinDC identification and further promoting the development of intelligent constitution identification. We also showed the importance ranking of the indicators related to YinDC through model interpretation methods and providing clinical guidance for individualized constitution identification. Using our proposed model, we achieved satisfying prediction results with fewer genetic indicators and could comprehensively reduce the patients’ economic burdens. With interpretation tool, the evidence of individual patient risk could be provided.

The rest of this paper is organized as follows, the methods section described the data sources and analysis methods. In the results section, four machine learning methods were used to construct prediction models, the differences between the models were compared, and the interpretability by SHAP of the models was provided. Lastly, the discussion section explained the results of the study from TCM constitution perspectives and methodology, and detailed the future research directions.

## Methods

### Participants

**Fig. 1:**
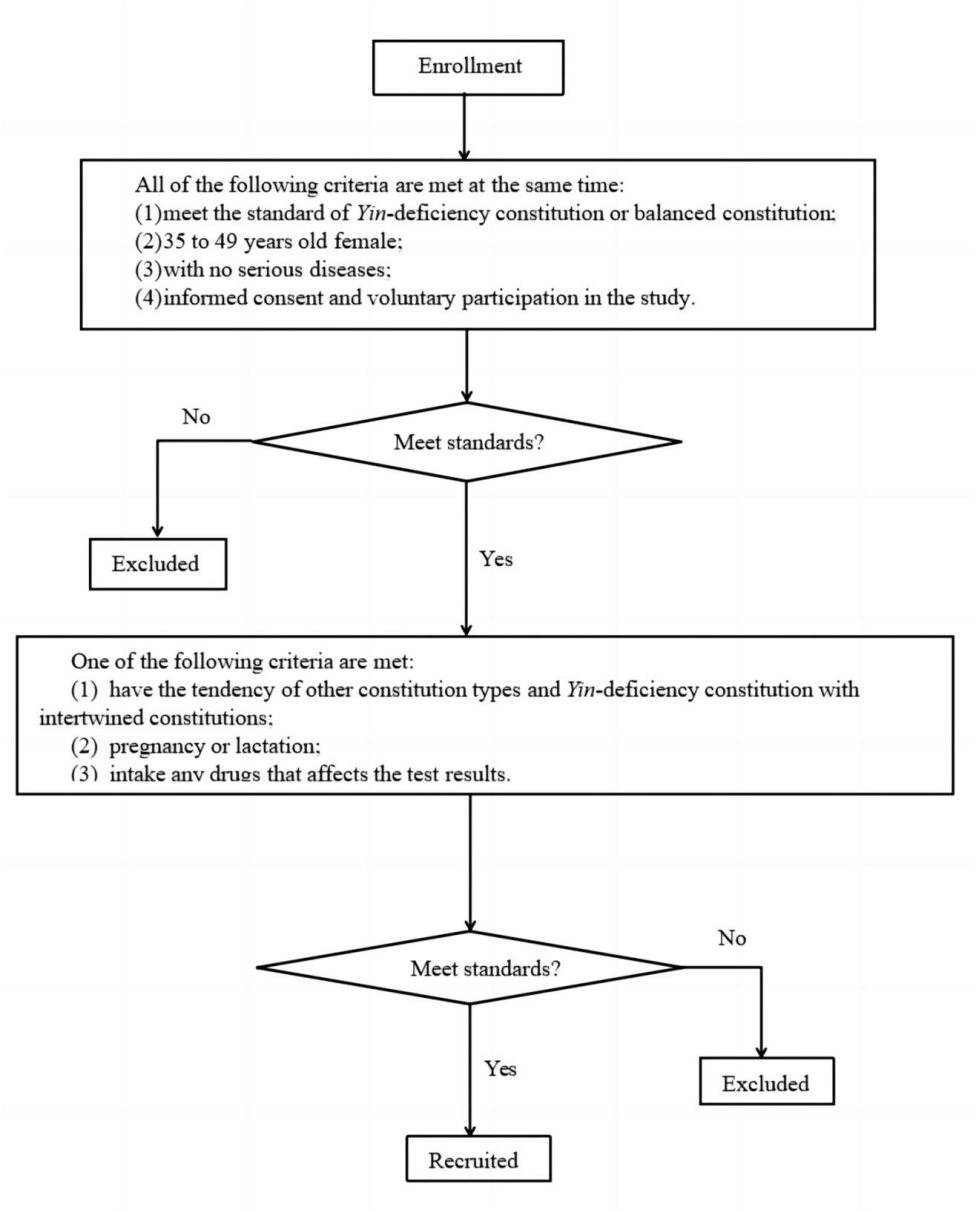
The flowchart of inclusion and exclusion criteria

Female volunteers aged 35–49 in Beijing were recruited for this study. Following TCM constitution identification, a total of 60 females were enrolled in the study, including 30 cases of YinDC and 30 cases of BC. The criteria determining YinDC and BC were based on the Standards for Classification and Judgment of TCM Constitution (published in 2009) [27]. A flowchart for the inclusion and exclusion criteria was plotted in Fig. 1.

The inclusion criteria include the following: (1) meet the standard of YinDC or BC; (2) female, 35 to 49 years old; (3) no history of serious diseases; (4) informed consent and voluntary participation in the study.

The exclusion criteria include the following: (1) have the tendency of other constitution types or YinDC with intertwined constitutions; (2) pregnancy or lactation; (3) taking medication that affects the test results.

### Blood genetic test

All participants were informed of the uniform collection location the day before blood sampling. 5 ml of fasting venous blood sample was collected from each participant at 8 a.m. Participants should abstain from alcohol and avoid exertion and menstruation before blood collection. To isolate total RNA, the blood samples were pretreated with Trizol reagent (Aidlab Biotechnologies Co., Ltd of Beijing, China). 3.7 μg of total RNA was reverse-transcribed using the HiScript Reverse Transcription Kit (Vazyme Biotech Co., Ltd. of Nanjing, China) under reaction conditions of 25 ºC for 5 min, 50 ºC for 15 min, 85 ºC for 5 min, and 4 ºC for 10 min. qRT-PCR analysis was performed with the cDNA and primers using SYBR Green Real-Tine OCR Master Mix (Applied Biosystems of MA, US) to assess the relative expression levels of mRNAs encoding TAK1, NFKBIA, CCL4, BCL2A1 and IL-8 under reaction conditions of 50 ºC for 2 min, 95 ºC for 10 min, 95 ºC for 30 s, and 60 ºC for 10 s [28].

### Analytical method

R statistical software was used for statistical analysis and plotting. Baseline data was expressed as mean ± standard deviation. Four machine learning methods, logistic regression, random forest, SVM, and XGBoost, were developed to construct prediction models of YinDC. The machine learning based models were built by R 4.0.3 for logistic regression, packages random forest 4.6–14, packages e1071 1.7-2, and XGBoost 1.7.5.1. The least absolute shrinkage and selection operator (LASSO) [29] regression model was utilized to screen the variables when constructing the logistic models. In addition to incorporating TAK1, NFKBIA, CCL4, BCL2A1, and IL-8 into the predictive models, the age and weight of the participants were taken into the modeling for real world data analysis.

The method of multi-fold cross-validation was used to evaluate each machine learning method. In the process of multi-fold cross-validation, the data set was divided into a training set and a test set. The models is trained with the baseline variables of participants on the training set, and prediction is made on the test set. The receiver operating characteristics (ROC) curve and area under curve (AUC) were used for model evaluation, developed by R package pROC 1.18.0. The cut-off value, diagnostic sensitivity, and specificity of prediction models were calculated. P < 0.05 was considered statistically significant. The AUC of different models in predicting YinDC were compared. Furthermore, the SHAP interpretation method was developed by R package shapviz 0.9.2.

## Results

### Comparison of baseline data

We analyzed the baseline data of all 60 participants in Table 1. The mean age of the YinDC group was 39.27 ± 3.77, while the mean age of the BC group was 44.27 ± 3.59. The age of the two groups showed a significant difference (P < 0.05), indicating that the YinDC group was younger than the BC group. There was no statistical difference in weight between the two groups.

**Table 1:** Baseline characteristics of balanced constitution and *Yin*-deficiency constitution groups

	BC group (n = 30)	YinDC group (n = 30)	P value
Age (years)	44.27 ± 3.59	39.27 ± 3.77	< 0.05*
Weight (kg)	55.40 ± 5.70	53.50 ± 5.44	≥ 0.05
TAK1	1.11 ± 0.75	1.16 ± 1.12	≥ 0.05
IL-8	4.54 ± 9.50	6.75 ± 14.43	≥ 0.05
NFKBIA	0.91 ± 0.89	3.93 ± 7.39	< 0.05*
CCL4	1.88 ± 1.78	3.95 ± 3.67	< 0.05*
BCL2A1	4.99 ± 7.18	0.93 ± 1.04	< 0.05*

^*^P < 0.05

An analysis of the differences of the five genes between two groups showed that there was a significant up-regulation in NFKBIA mRNA and CCL4 mRNA expression and a considerable down-regulation in BCL2A1 mRNA expression in the YinDC group compared with the BC group (P < 0.05). However, the two groups had no statistically significant difference in TAK1 mRNA and IL-8 mRNA expressions (P > 0.05).

### Screening of genetic predictors for *Yin*-deficiency constitution

**Fig. 2:**
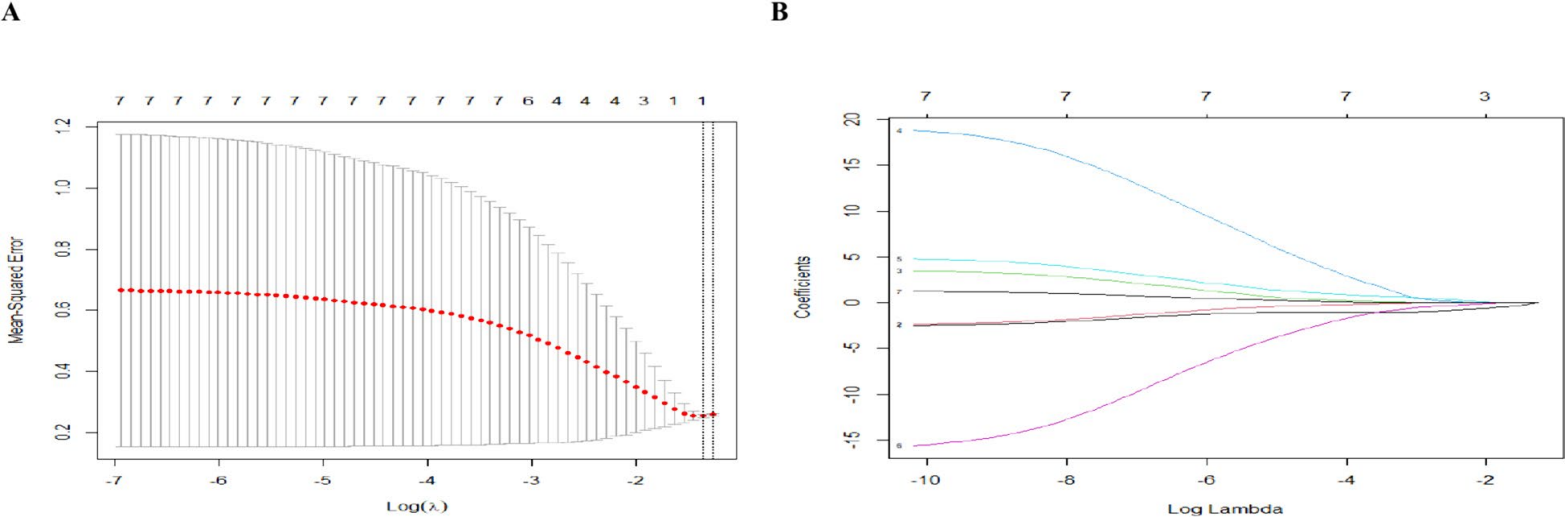
LASSO regression analysis was used to select predictors. **A** The optimal parameter lambda in LASSO regression was determined through cross-validation. The lambda corresponding to the mean-square error (MSE) was selected as the optimal value. **B** The cross-validation to draw vertical lines at selected values

LASSO regression analysis was conducted on the remaining independent variables, see Fig. 2. We identified independent predictive features in the training set by nonzero coefficients in the LASSO regression and selected the optimal parameter lambda through threefold cross-validation. The results showed that there were four variables left, including age, NFKBIA, BCL2A1 and CCL4, when lambda was 0.1. The selected genetic indicators were consistent with that in the previous studies, confirming that NFKBIA, BCL2A1 and CCL4 are related to YinDC. In particular, the coefficient of three genetic indicators in the regression model were 0.014, 0.042 and -0.047, indicating that NFKBIA and CCL4 gene expressions increased, while BCL2A1 gene expression decreased among YinDC.

### Comprehensive analysis of classified models with multiple genetic indicators

We constructed multi-variable prediction models for YinDC classification using four machine learning methods, including logistic regression, random forest, SVM, and XGBoost. Based on each machine learning method, we constructed 11 multi-variable prediction models (M1–M11) with different combinations of five genetic indicators. Thus, this study conducted a total of 44 YinDC multi-variable prediction models. The performance of the prediction models was evaluated using the AUC values, which refer to the area under the receiver operating characteristic (ROC) curve.

**Fig. 3:**
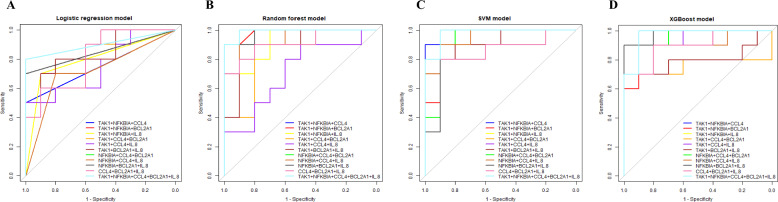
The ROC curves of prediction model by four machine learning methods, **A** logistic regression, **B** random forest, **C** support vector machine, **D** XGBoost

Logistic regression, random forest, SVM, and XGBoost and three-fold cross validation were performed on the training set and the performances on the test set were shown. Figure 3 presented of the total ROC curves all prediction models with combined genetic indicators. The best AUC of models in logistic regression, random forest, SVM, and XGBoost were 0.930 (95% CI 0.839–1.000), 0.937 (95% CI 0.844–1.000), 0.940 (95% CI 0.848–1.000), and 0.905 (95% CI 0.794–0.995). We also provided their positive predictive value (PPV) and they were 0.970, 0.909, 0.939 and 1.000. AUC, sensitivity, specificity and their 95% CI are detailed in Table 2. P values of most models indicated that the predictive models were significant.

**Table 2:** Validation result of different prediction models using logistic regression, random forest, support vector machine and XGBoost methods

	M1	M2	M3	M4	M5	M6	M7	M8	M9	M10	M11
Logistic regression											
AUC	0.897**	0.930***	0.860**	0.870**	0.820	0.847**	0.920***	0.890*	0.890**	0.840**	0.877**
95% CI	0.798–0.971	0.839–1.000	0.705–0.993	0.710–1.000	0.665–0.975	0.663–1.000	0.824–1.000	0.779–0.980	0.728–1.000	0.653–1.000	0.733–0.994
Cut-off	0.525	0.562	0.440	0.388	0.493	0.522	0.473	0.674	0.541	0.252	0.167
Sensitivity	0.833	0.900	0.900	0.900	0.833	0.800	0.900	0.900	0.900	0.900	0.867
95% CI	0.730–0.937	0.805–0.995	0.805–0.995	0.755–1.000	0.730–0.937	0.561–0.995	0.805–0.995	0.805–0.995	0.805–0.995	0.755–1.000	0.701–1.000
Specificity	0.933	0.967	0.767	0.767	0.833	0.867	0.933	0.867	0.900	0.733	0.900
95% CI	0.809–1.000	0.905–1.000	0.521–0.968	0.521–0.968	0.730–0.937	0.701–1.000	0.851–1.000	0.660–1.000	0.755–1.000	0.485–0.937	0.755–1.000
Random forest											
AUC	0.910**	0.898**	0.882**	0.793*	0.753	0.790	0.937**	0.913***	0.913**	0.877**	0.908**
95% CI	0.779–1.000	0.775–1.000	0.729–1.000	0.568–0.990	0.522–0.972	0.574–0.983	0.844–1.000	0.790–1.000	0.795–1.000	0.716–1.000	0.794–1.000
Cut-off	0.643	0.483	0.597	0.566	0.428	0.455	0.598	0.650	0.558	0.462	0.474
Sensitivity	0.833	0.900	0.867	0.867	0.867	0.867	0.870	0.833	0.867	0.900	0.933
95% CI	0.615–0.995	0.714–1.000	0.701–1.000	0.710–0.995	0.660–1.000	0.660–1.000	0.755–1.000	0.606–1.000	0.660–1.000	0.714–1.000	0.809–1.000
Specificity	0.933	0.833	0.867	0.800	0.633	0.733	0.900	0.933	0.933	0.800	0.800
95% CI	0.851–1.000	0.656–0.995	0.710–0.995	0.552–1.000	0.341–0.926	0.467–0.968	0.720–1.000	0.809–1.000	0.851–1.000	0.552–1.000	0.561–0.995
SVM											
AUC	0.897**	0.937**	0.860*	0.837**	0.830*	0.840**	0.923**	0.900**	0.927***	0.860**	0.940***
95% CI	0.776–0.998	0.845–1.000	0.702–0.998	0.655–0.996	0.661–0.987	0.655–1.000	0.820–1.000	0.774–1.000	0.807–1.000	0.679–1.000	0.848–1.000
Cut-off	0.488	0.614	0.551	0.561	0.488	0.455	0.534	0.592	0.619	0.455	0.581
Sensitivity	0.867	0.900	0.900	0.767	0.833	0.867	0.900	0.833	0.900	0.900	0.900
95% CI	0.710–0.995	0.805–0.995	0.805–0.995	0.521–0.968	0.730–0.937	0.660–1.000	0.805–0.995	0.670–0.968	0.805–0.995	0.755–1.000	0.805–0.995
Specificity	0.900	0.967	0.800	0.900	0.767	0.800	0.933	0.933	0.933	0.767	0.933
95% CI	0.714–1.000	0.905–1.000	0.575–0.970	0.760–0.968	0.581–0.937	0.552–1.000	0.851–1.000	0.809–1.000	0.809–1.000	0.581–0.937	0.809–1.000
XGBoost											
AUC	0.905**	0.882**	0.888**	0.770	0.837	0.772	0.878**	0.898**	0.892**	0.835*	0.895**
95% CI	0.794–0.995	0.736–0.993	0.748–0.993	0.532–1.000	0.663–0.985	0.543–0.984	0.738–0.995	0.772–0.995	0.763–0.993	0.655–0.993	0.770–0.995
Cut-off	0.740	0.699	0.750	0.620	0.483	0.432	0.615	0.724	0.743	0.331	0.555
Sensitivity	0.800	0.767	0.767	0.767	0.833	0.767	0.767	0.800	0.767	0.867	0.833
95% CI	0.635–0.937	0.539–0.937	0.539–0.937	0.571–0.963	0.670–0.968	0.515–0.989	0.539–0.937	0.635–0.937	0.539–0.937	0.710–0.995	0.730–0.937
Specificity	1.000	0.967	1.000	0.833	0.867	0.800	0.900	0.967	0.967	0.733	0.900
95% CI	1.000–1.000	0.905–1.000	1.000–1.000	0.730–0.937	0.710–0.995	0.611–0.989	0.755–1.000	0.905–1.000	0.905–1.000	0.467–0.968	0.755–1.000

M1: “TAK1 + NFKBIA + CCL4”, M2: “TAK1 + NFKBIA + BCL2A1”, M3: “TAK1 + NFKBIA + IL-8”, M4: “TAK1 + CCL4 + BCL2A1”, M5: “TAK1 + CCL4 + IL-8”, M6: “TAK1 + BCL2A1 + IL-8”, M7: “NFKBIA + CCL4 + BCL2A1”, M8: “NFKBIA + CCL4 + IL-8”, M9: “NFKBIA + BCL2A1 + IL-8”, M10: “CCL4 + BCL2A1 + IL-8”, M11: “TAK1 + NFKBIA + CCL4 + BCL2A1 + IL-8”, and two covariates, patients’ age and weight, were added to M1-M11

^*^P < 0.05

**P < 0.01

***P < 0.001

The AUC values of all models in logistic regression were not less than 0.820. The best AUC of models in logistic regression was 0.930 (M2: TAK1, NFKBIA, and BCL2A1; 95% CI 0.839–1.000), with a cut-off of 0.562, a sensitivity of 0.900 (95% CI 0.805–0.995), and a specificity of 0.967 (95% CI 0.905–1.000). Besides, the positive predictive value of M2 reached 0.970, further affirming the predictive accuracy of the model. These results indicated that the prediction model constructed by logistic regression had good predictive accuracy.

As for the models constructed by random forest, five prediction models had an AUC higher than 0.900. The best AUC was topped at 0.937 (M7: NFKBIA, CCL4, and BCL2A1; 95% CI 0.844–1.000), and PPV was 0.909. Compared with other machine learning models, the models by random forest showed smaller size of confidence intervals indicating the model stability and achieved high accuracy by using fewer prediction indicators.

All the prediction models by SVM had statistical significance. M11, composed of all five gene indicators TAK1, NFKBIA, CCL4, BCL2A1, and IL-8, corresponded to the best AUC of 0.940 (95% CI 0.848–1.000) with the cut-off of 0.581, the sensitivity of 0.900 (95% CI 0.805–0.995), the specificity of 0.933 (95% CI 0.809–1.000), and PPV of 0.939. Among all the models in the study, the M11 by SVM had the best AUC. However, it had limitation that the model was based on all five gene indicators, which represented higher medical costs.

Table 2 also shows the results of model by XGBoost. About 1/3 of the prediction models did not show statistical significance. The best AUC of model by XGBoost was 0.905 (M1: TAK1, NFKBIA, and CCL4; 95% CI 0.794–0.995), which was not superior to the models constructed by which was not superior to the models constructed by other three machine learning models.

**Fig. 4:**
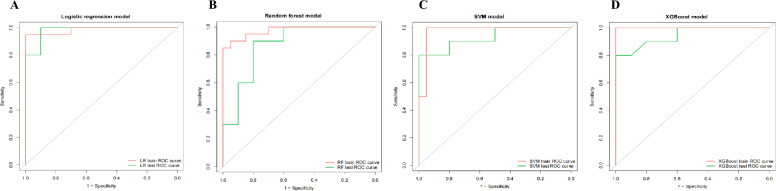
ROC curves on training and test dataset of the optimal prediction models constructed by the four machine learning methods

Furthermore, we plotted the ROC curves based on the training set and test set of the optimal models constructed by the four machine learning methods respectively (Fig. 4A–D). As presented in the figures, the areas under the ROC curves of the training set were all larger than those of the test set, which is consistent with the conventional cognition. The AUC values of the ROC curves in the training set were 0.985 for LR, 0.975 for RF, 0.975 for SVM, and 1.000 for XGBoost. In the test set, the AUC values for the LR model were 0.980, 0.870 for RF, 0.930 for SVM, and 0.945 for XGBoost. The AUC values of the training and test sets in all four models were not less than 0.870, showing good prediction performance.

To sum up, M11 of SVM containing all the five gene indicators had the best AUC of 0.940. While, M7 of random forest containing NFKBIA, CCL4, and BCL2A1 was only slightly inferior to M11 of SVM, with an AUC of 0.937. Clinicians will have to consider two additional genetic indicators, TAK1 and IL-8, to predict YinDC with SVM. Based on the above comprehensive analysis, considering the efficiency and cost of clinical diagnosis and treatment, the model by random forest is more valuable and generalizable which was recommended as the optimal model.

### Hypothesis testing results of comparing models

We selected the optimal model of each machine learning method and five sub-optimal models by the AUC and conducted hypothesis tests. The results are presented in Table 3. It was found that there was no significant difference between the optimal prediction model and the sub-optimal models in logistic regression, random forest, SVM, and XGBoost. This indicates that the optimum model did not have a significant advantage over sub-optimal models.

**Table 3:** Comparison of the differences of AUC in the prediction models

	Index	M2 vs M7	M2 vs M1	M2 vs M8	M2 vs M9	M2 vs M11	M2 vs M4
Logistic regression	AUC	0.930/0.920	0.930/0.897	0.930/0.890	0.930/0.890	0.930/0.877	0.930/0.870
	P value	0.519	0.794	0.094	0.409	0.306	0.607

	Index	M7 vs M8	M7 vs M9	M7 vs M1	M7 vs M11	M7 vs M2	M7 vs M3
Random forest	AUC	0.937/0.913	0.937/0.913	0.937/0.910	0.937/0.908	0.937/0.898	0.937/0.882
	P value	0.350	0.189	0.480	0.230	0.381	0.190

	Index	M11 vs M2	M11 vs M9	M11 vs M7	M11 vs M8	M11 vs M1	M11 vs M3
SVM	AUC	0.940/0.937	0.940/0.927	0.940/0.923	0.940/0.900	0.940/0.897	0.940/0.860
	P value	0.591	0.623	0.144	0.677	0.604	0.950

	Index	M1 vs M8	M1 vs M11	M1 vs M9	M1 vs M3	M1 vs M2	M1 vs M7
XGBoost	AUC	0.905/0.898	0.905/0.895	0.905/0.892	0.905/0.888	0.905/0.882	0.905/0.878
	P value	1.000	0.902	0.658	0.202	0.473	0.690

### Identification of YinDC features based on machine learning

**Fig.5:**
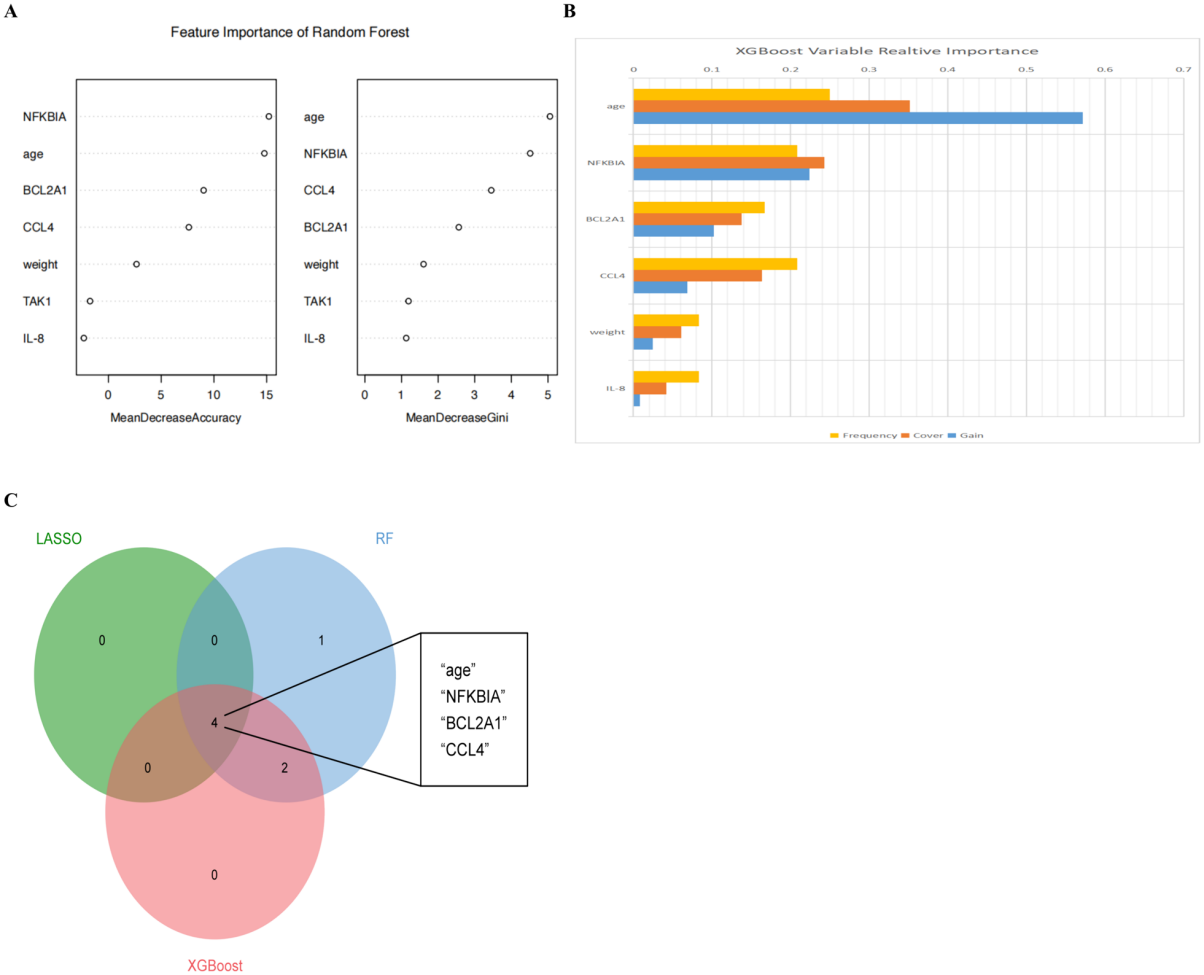
Identification of YinDC features. **A** Importance of variables selection using the RF method. **B** Important variables screened by the XGBoost method. **C** Venn diagram showing the features intersected by LASSO, RF, and XGBoost

While screening out the optimal predictive model, we hope to screen out the most relevant variables to the prediction of YinDC, to lead to more accurate research findings. High-importance features can be selected using the non-linear methods such as random forest or XGBoost. The importance ranking of each variable generated by the random forest and XGBoost methods was shown in Fig. 5A, B. We compared the results with the independent predictive features screened by LASSO regression and considered the overlapping metrics of the three machine learning methods to be the most relevant features for YinDC. The result was displayed in the visual form of a Wayne diagram in Fig. 5C. Interestingly, it was noteworthy that the important features screened were identical to the metrics used in the optimal model, further affirming the accuracy of the predictive model.

### Applying SHAP to model interpretation

**Fig. 6:**
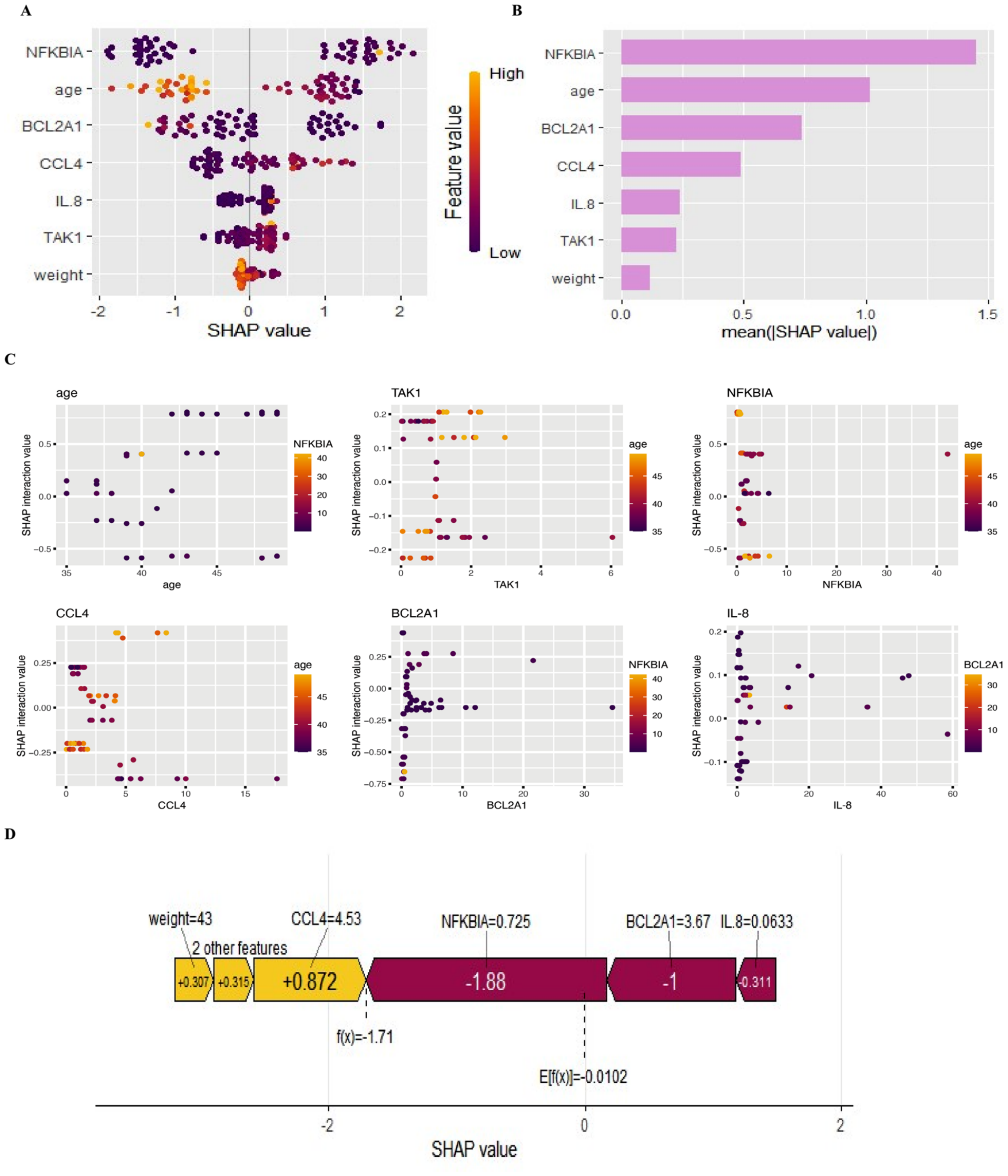
Model interpretation with SHAP. **A** SHAP summary plot of attributes of characteristics. Each line represents features, and the abscissa represents the SHAP value. Light dots represent higher eigenvalues and dark dots represent lower eigenvalues. **B** Feature significance ranking indicated by SHAP, describing the importance of each predictor in the development of the prediction model.**C** SHAP interaction values plots. **D** The SHAP value represents the predictors of individual patients and their contribution to the prediction of YinDC.

To provide a visual explanation on the significance of the predictors, we applied SHAP to evaluate the contribution of predictors in YinDC prediction. A SHAP summary plot was plotted to illustrate the most important predictor in developing the prediction model, see Fig. 6A. Each point in the figure represents an individual sample, and light dots represent high risk values and dark dots represent low risk values. Larger absolute SHAP values represent a greater influence on the model prediction. The SHAP feature import barplot further visually exhibits the contribution levels of each feature, as shown in Fig. 6B. NFKBIA, age, BCL2A1, and CCL4 were the most important predictors of the model. As the previous study found that age is associated with TCM constitution [5], age can be regarded as an important predictor for YinDC prediction in the real world.

The contribution of predictors interactions to the model’s prediction was visualized by calculating the SHAP interaction values (Fig. 6C). All computed SHAP interaction values were grouped based on whether one of the predictors used in each interaction calculation has a high or low value. For instance, in the first image in Fig. 6C, the x-axis represents the values of age after robust scaling, and the y-axis shows the calculated SHAP interaction values between age and NFKBIA, reflecting the impact of their interactions on the model’s prediction for each sample.

In addition, we provided a typical example to illustrate the interpretability of the model, see Fig. 6D. Among the five predictors of this subject, NFKBIA, BCL2A1, and CCL4 had the most contribution to the predictive mortality. The plot reflects the individual differences of importance among predictors of the same TCM constitution. Based on the above, SHAP well-explained the individual important predictors and could provide practical guidance for clinical constitution identification.

Each of the four machine learning methods has advantages and characteristics. Among all constructed prediction models, the SVM-constructed M11 with all five genetic indicators TAK1, NFKBIA, CCL4, BCL2A1, and IL-8 had the highest accuracy of 0.940. At the same time, the accuracy of random forest-constructed M7 scored second with 0.937 with two less indicators, using NFKBIA, CCL4, and BCL2A1. For the other two machine learning methods, logistic regression-constructed models had good interpretability and stability, and XGBoost-constructed models had moderate accuracy and sensitivity, but they did not stand out from the other models.

Overall, we constructed YinDC prediction models based on four machine learning methods and got pretty performance. The models of logistic regression, random forest, and XGBoost were not significantly improved when we added the feature indicators to predict YinDC. While, predicting with fewer indicators to achieve an ideal prediction model is more desirable. Therefore, random forest with three genetic indicators (NFKBIA, CCL4, and BCL2A1) is considered applicable in clinical practice.

## Discussion

Combining traditional Chinese medicine theories with objectivized and modernized methods and indicators is conducive to the construction of a comprehensive evaluation system with the characteristics of Chinese medicine. TCM constitution provides a suitable carrier and scientific guidance for proactive health. Applying advanced machine learning techniques is essential in TCM, which could help establish dependable theories and modern medical techniques. This enables us to better integrate proactive health concepts such as constitution identification into all aspects of healthcare, including prevention and treatment of disease [30]. This study combines TCM theories with objectivized indicators, and the findings further corroborate the correctness of the theories. Based on the YinDC prediction models, we could generalize the model into other constitutions, providing a research pattern for TCM constitution identification.

In this study, we constructed multi-variable models based on machine learning methods to predict YinDC. Among all the prediction models, the M11 of SVM (TAK1, NFKBIA, CCL4, BCL2A1, and IL-8) topped with AUC of 0.940, followed by M7 of random forest (TAK1, NFKBIA, and BCL2A1) with AUC of 0.937. When the performance of models was almost close, the clinical practice is equally important. Therefore, the model of random forest is more valuable in clinical predictions. Additionally, we used SHAP to explain the contribution of the features to model predictions, providing the interpretability for machine learning models.

The genetic indicators NFKBIA, CCL4 and BCL2A1 screened in this study, which are closely related to the prediction of YinDC, are upstream and downstream genes of the Nuclear factor-kappaB (NF-kappaB) signaling pathways. NF-kappaB plays important roles in diverse biological processes by regulating the expression of a large number of target genes that are involved in the immune and inflammatory response, cell proliferation and survival [31]. We can learn that the differential expression of these genes plays a role in the apoptosis of immune cells in YinDC. YinDC is characterized by dryness and heat, which is associated with the body being in a state of chronic inflammation. The feature genes screened in this study are precisely associated with inflammatory response, which can well explain the physical characteristics of YinDC.

There were some limitations in this study. Firstly, the genes selected for this study were commonly involved in the NF-κB activation pathway regulation. Whether other genes remains to be further verified. Secondly, the prediction model may be constrained by the smaller sample size. Further, we will generalize the model into other TCM constitution types and more population. To demonstrate reproducibility, further prospective studies in multiple centers with large sample sizes will be developed.

## Conclusion

This study screened features related to YinDC prediction using the LASSO regression. Logistic regression, random forest, SVM and XGBoost were used to construct YinDC prediction models. SHAP method was developed for the machine model interpretation of important genetic indicators. The optimal model was identified with pretty performance and clinical value, providing the guidance for the clinical practice and generalized pattern for TCM constitution identification.

## Data Availability

The datasets used and analysed during the current study are available from the corresponding author on reasonable request.

## Abbreviations

TCM: Traditional Chinese Medicine
YinDC: *Yin*-deficiency constitution
AI: Artificial intelligence
ML: Machine learning
TAK1: TGF beta-activated kinase 1
NFKBIA: NFKB inhibitor alpha
CCL4: Chemokine CC motif ligand 4
BCL2A1: BCL2 related protein A1
IL-8: Interleukin-8
SVM: Support vector machine
XGBoost: EXtreme Gradient Boosting
SHAP: Shapley Additive exPlanations
LASSO: Least absolute shrinkage and selection operator
ROC: Receiver operating characteristic
AUC: Area under curve
